# In vivo three-dimensional evaluation of tumour hypoxia in nasopharyngeal carcinomas using FMT-CT and MSOT

**DOI:** 10.1007/s00259-019-04526-x

**Published:** 2019-11-08

**Authors:** Wenhui Huang, Kun Wang, Yu An, Hui Meng, Yuan Gao, Zhiyuan Xiong, Hao Yan, Qian Wang, Xuekang Cai, Xin Yang, Bin Zhang, Qiuying Chen, Xing Yang, Jie Tian, Shuixing Zhang

**Affiliations:** 1 Medical Imaging Center, the First Affiliated Hospital, Jinan University, No. 163, Huangpu West Road, Tianhe District, Guangzhou, Guangdong 510632 China; 2 CAS Key Laboratory of Molecular Imaging, Institute of Automation Chinese Academy of Sciences, No. 95 Zhongguancun East Road, Haidian District, Beijing, 100190 China; 3 Beijing Advanced Innovation Center for Big Data-Based Precision Medicine, Beihang University, Beijing, 100191 China; 4 Department of Chemical and Bio-molecular Engineering, The university of Melbourne, Melbourne, Victoria 3010 Australia; 5 Engineering Laboratory for Functionalized Carbon Materials, Graduate School at Shenzhen, Tsinghua University, Shenzhen, 518055 China; 6 Department of Diagnostic Imaging, National Cancer Center/Cancer Hospital, Chinese Academy of Medical Sciences and Peking Union Medical College, Beijing, 100021 China; 7 Department of Nuclear Medicine, Peking University First Hospital, No. 8 Xishiku Road, Xicheng District, Beijing, 100034 China

**Keywords:** Nasopharyngeal carcinoma, Tumour hypoxia, Fluorescence molecular tomography, Multispectral optoacoustic tomography, Carbonic anhydrase IX

## Abstract

**Purpose:**

Accurate evaluation of hypoxia is particularly important in patients with nasopharyngeal carcinoma (NPC) undergoing radiotherapy. The aim of this study was to propose a novel imaging strategy for quantitative three-dimensional (3D) evaluation of hypoxia in a small animal model of NPC.

**Methods:**

A carbonic anhydrase IX (CAIX)-specific molecular probe (CAIX-800) was developed for imaging of hypoxia. Mouse models of subcutaneous, orthotopic, and spontaneous lymph node metastasis from NPC (5 mice per group) were established to assess the imaging strategy. A multi-modality imaging method that consisted of a hybrid combination of fluorescence molecular tomography-computed tomography (FMT-CT) and multispectral optoacoustic tomography (MSOT) was used for 3D quantitative evaluation of tumour hypoxia. Magnetic resonance imaging, histological examination, and immunohistochemical analysis were used as references for comparison and validation.

**Results:**

In the early stage of NPC (2 weeks after implantation), FMT-CT enabled precise 3D localisation of the hypoxia biomarker with high sensitivity. At the advanced stage (6 weeks after implantation), MSOT allowed multispectral analysis of the biomarker and haemoglobin molecules with high resolution. The combination of high sensitivity and high resolution from FMT-CT and MSOT could not only detect hypoxia in small-sized NPCs but also visualise the heterogeneity of hypoxia in 3D.

**Conclusions:**

Integration of FMT-CT and MSOT could allow comprehensive and quantifiable evaluation of hypoxia in NPC. These findings may potentially benefit patients with NPC undergoing radiotherapy in the future.

**Graphical abstract:**
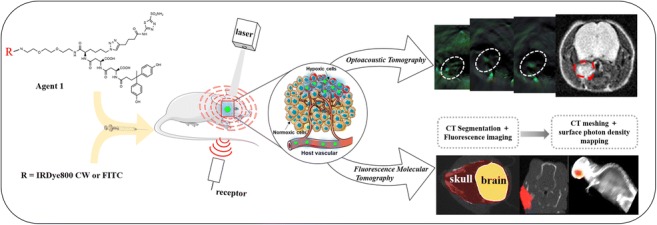
A novel multimodality imaging strategy for three-dimensional evaluation of tumour hypoxia in an orthotopic model of nasopharyngeal carcinoma.

**Electronic supplementary material:**

The online version of this article (10.1007/s00259-019-04526-x) contains supplementary material, which is available to authorized users.

## Introduction

Tumour hypoxia plays a crucial role in tumour aggressiveness, metastasis, resistance to chemoradiotherapy, and increased rate of recurrence [1–3]. It is widely considered to be an independent predictor of a poor prognosis in most types of tumours, and especially in nasopharyngeal carcinoma (NPC) [4–6]. NPC is a common malignancy in East and South-east Asia and is primarily treated by radiotherapy because of its awkward location and high sensitivity to radiotherapy [7]. However, hypoxia-induced resistance to radiation remains a serious obstacle to achieving optimal outcomes in patients with locally advanced NPC [8]. Guiding hypoxia-targeted therapy in individual patients continues to be a clinically relevant problem, and there is an unmet demand for a non-invasive method to visualise and evaluate hypoxia in NPC.

Hypoxia arises in tumours because of an imbalance between oxygen supply and consumption [9]. This characteristic means that a higher radiation dose is needed in the hypoxic region to counteract its high resistance to treatment, while applying a smaller radiation dose in the non-hypoxic area to minimise side effects. Wider application of intensity-modulated radiotherapy in recent times has made it possible to regulate the dose distribution more precisely. Therefore, accurate assessment of tumour hypoxia is critical when planning individualised radiotherapy. Various non-invasive imaging techniques, including positron emission tomography(PET)/computed tomography(CT), planar optical imaging, and magnetic resonance imaging (MRI), have been used to monitor tumour hypoxia [10–13]. Among them, PET/CT is a preferable modality in preclinical and clinical researches of head and neck cancer, because it can directly identify the presence of hypoxia with high sensitivity and three-dimensional (3D) quantification ability [13–15]. However, tumour hypoxia is highly heterogeneous, and there is still no conclusive knowledge about the evolution of tumour hypoxia during the disease progression or treatment with the existing imaging strategies [16]. Therefore, development of a novel imaging strategy to allow the 3D visualisation and quantification of hypoxia in NPC while assuring sufficient sensitivity and accuracy presents a challenge.

Fluorescence molecular tomography (FMT) is a novel optical molecular imaging technique that can reconstruct the biodistribution of fluorescence probes in animal models in 3D [17–19]. It converts the conventional qualitative and planar fluorescence molecular imaging (FMI) into a quantitative approach. FMT is frequently combined with CT, so that regions with high probe accumulation can be merged into anatomical structures for visualisation [20]. This unique ability holds great promise for in vivo quantitative imaging of the hypoxia-specific fluorescence probe distribution in NPC.

Unlike FMT-CT, multispectral optoacoustic tomography (MSOT) combines the compelling features of both light and sound and has a dynamic imaging ability that allows it to obtain exogenous and endogenous information, such as the distribution of optoacoustic probes and haemoglobin molecules in 3D [21, 22]. Furthermore, its spectral separation enables the visualisation of oxygenated haemoglobin (HbO_2_) and deoxygenated haemoglobin (Hb) simultaneously, which has been used to investigate tumour hypoxia indirectly [23]. We hypothesized that integration of the quantifiable information acquired from both FMT-CT and MOST would be a comprehensive imaging strategy allowing accurate evaluation of hypoxia in NPC.

We chose carbonic anhydrase IX (CAIX), a secondary marker of hypoxia, as our cellular marker when designing the hypoxia targeting probe. CAIX is primarily upregulated by the activated hypoxia-inducible factor-1α (HIF-1α) [24, 25]*.* It is confirmed to be a biomarker of hypoxia in head and neck tumours and a robustly negative prognostic marker for NPC [4, 5, 26, 27]. As a transmembrane protein, CAIX is also relatively easier for molecular probes to access in vivo. A recent study reported the discovery of a 4,4-bis(4-hydroxyphenyl) valeric acid/acetazolamide-based dual-motif CAIX inhibitor with significantly improved affinity [28], which inspired us to adapt it for use in our study, and an IRDye 800CW-conjugated probe (CAIX-800) was synthesised.

In this study, we aimed to combine the strengths of FMT and MSOT by applying a hypoxia-targeting optical probe in order to achieve accurate non-invasive 3D quantification of hypoxia in NPC-bearing mouse models. This multi-modality hypoxia imaging strategy was intensively evaluated in subcutaneous, orthotopic, and spontaneous nodal metastasis mouse models. The strengths and weaknesses of each modality, as well as the benefits of combining both approaches, in the evaluation of hypoxia in NPC were illustrated in different perspectives. MRI, histological analysis, and immunohistochemical analysis were used for comparison and validation. To our knowledge, orthotopic and nodal metastasis mouse models of NPC have not been investigated by FMT-CT and MSOT before.

## Materials and methods

### Synthesis and characterization of CAIX-800

All solvents and chemicals were obtained from commercial sources and used without further purification. CAIX targeting agent **1** was synthesised in the laboratory as previously described [29]. IRDye 800 N-hydroxysuccinimide ester (NHS) (Biotium Inc., LI-COR, Fremont, CA) and agent **1** were conjugated following a previously reported method [30]. Briefly, agent **1**, IRDye 800 NHS, and trimethylamine (in a 1:1:6 M ratio) were mixed in dimethylformamide and stirred at room temperature for 2 h. After the solvent was removed under vacuum, the product was purified by high-performance liquid chromatography. CAIX-800 was purified using an Inertsil C18 Luna 46 × 150-mm column on a 1260 Infinity LC system (Agilent, Santa Clara, CA). Mass spectroscopy was used to characterise the conjugates of the probe. The CAIX-FITC used for the in vitro cell binding assay was prepared and tested in the same way as CAIX-800.

### Cell culture and cellular uptake of CAIX-FITC in vitro

Two cell lines, i.e., 5-8F (a CAIX positive control [31]) and C666-1 (a CAIX negative control [32]) were provided by Southern Medical University. The cells were cultured at 37 °C and 5% CO_2_ in Dulbecco’s modified Eagle’s medium supplemented with 10% foetal bovine serum and with penicillin and streptomycin (Gibco Invitrogen, Carlsbad, CA, USA). The cells were incubated on confocal plates (2 × 10^5^ cells/plate) for 24 h. After removal of the medium, CAIX-FITC and free FITC were incubated separately with the cells for 4 h at a final concentration of 10 nM. The cells were then washed with phosphate-buffered saline three times and fixed with 4% paraformaldehyde for 15 min at 37 °C. The cytoskeleton was first stained with rhodamine phalloidin for 30 min and the nucleus was stained with 15 μg/ml of DAPI (4, 6-diamidino-2-phenylindole) for another 8 min at room temperature. All images were acquired using a confocal laser scanning microscope (LSM-710, Carl Zeiss, Oberkochen, Germany). Imaging processing was performed using ZEN 2.3 lite (Zeiss, Germany). Fluorescence quantification was analyzed using ImageJ 2.X (LOCI, University of Wisconsin).

### Creation of animal models

Four-week-old male BALB/c nude mice (Vital River Laboratory Animal Technology Co. Ltd., Beijing, China) were acclimated for 1 week before the study. The animals were kept in a specific pathogen-free unit. All surgical procedures were performed using a sterile hood. Two types of tumour models, i.e., subcutaneous and orthotopic NPC xenografts, were established. Briefly, 200 μL of phosphate-buffered saline (0.01 mol/L, pH 7.2) containing a suspension of 1.8 × 10^6^ 5-8F cells or 1.2 × 10^7^ C666-1 cells were injected subcutaneously into the lower left flank (*n* = 5 mice per group). The experiments started when the tumour diameter was 6–8 mm, an average tumour volume of approximately 126 mm^3^. Orthotopic cells were implanted as previously described [33]. Five mice per cohort were injected with 25 μl of 1.1 × 10^5^ 5-8F-fLuc cells into the nasopharynx using a 28-gauge needle. The animals were allowed to recover postoperatively for 30 min on a heated pad. Orthotopic implantation was confirmed by whole-body fluorescence imaging (PhotonIMAGER; Biospace Lab, Nesles-la-Vallée, France). All mice received a weekly intraperitoneal injection containing 3 mg of luciferin bioluminescent substrate (D-luciferin potassium salt, Perkin Elmer, Waltham, MA, USA) 4 min before imaging to monitor growth of the orthotopic tumour. Luciferase activity was quantified using region of interest analysis of the entire tumour.

### MRI of orthotopic animal models

The orthotopic tumours were located by 1.5-T MRI (M3TM, Aspect Imaging, Shoham, Israel) using a head and neck protocol that included axial and coronal T2-weighted imaging with the following parameters: repetition time 6000 ms, echo time 50 ms, slice thickness 0.7 mm, and slice spacing 0.2 mm. During acquisition of the images, all mice were anaesthetised using an isoflurane-oxygen gas mixture (500 ml/min, Matrx VMR Small Animal Anesthesia Machine, Matrx, USA).

### Biodistribution and tumour selectivity of CAIX-800 in vivo

Subcutaneous mouse models were established to confirm the distribution and tumour selectivity of CAIX-800 using two different cell lines. The mice (5 per group) received either 200 μl of 15 μM CAIX-800 or equimolar quantities of IRDye 800 as the control by intravenous injection. The targeting efficiency of CAIX-800 was further illustrated by a blocking study. A blocking dose (15 μM) of free **1** (without fluorescent labeling) was injected 30 min before injection of CAIX-800. Each group was monitored from 0 to 24 h. To quantitatively analyse the fluorescence signal in the tumour area, the tumour-to-background ratio (TBR) was calculated by drawing regions of interest within the tumour area and the ipsilateral auricle of each mouse. The average fluorescence intensity of the tumour area was then divided by that of the ipsilateral auricle. To validate our findings, 3D volumetric optoacoustic imaging of the 5-8F and C666-1 tumours was performed at different time points (pre, 4, 8, 12, and 24 h) using a 384 ultrasound transducer with a centre frequency of 10 MHz organised in a hemispherical array with a radius of curvature of 4 cm.

At 24-h post-injection, the mice were euthanised by cervical dislocation. The heart, lungs, pancreas, spleen, liver, kidneys, small intestines, and tumours were then collected for ex vivo fluorescence imaging. The harvested tumours were immediately snap-frozen at − 80 °C in a deep freeze. A series of sequential 10-μm-thick frozen sections were then cut. An inverted microscope (Leica, Wetzlar, Germany) was used to confirm the difference in fluorescence signals between the different types of tumour. The same frozen sections were then used for immunohistochemistry. A standard immunohistochemical protocol was used to stain the tissue sections with anti-CAIX antibody (1:100 dilution, ProteinTech Group Inc., Rosemont, USA), as described elsewhere [34].

### NPC-specific multimodality imaging of orthotopic mouse models

At 2-week post-implantation, the orthotopic mouse models (*n* = 5) were used for NPC-specific multimodality imaging. First, whole-body fluorescence imaging (PhotonIMAGER) was performed at different time points (2, 4, 6, 8, and 12 h) after intravenous injection of CAIX-800 with an excitation wavelength of 749 nm and a 776-nm filter. A video based on the fluorescence signal at the 8-h time point was reconstructed using a commercial M3 Vision system (PhotonIMAGER, Bio space Lab). MSOT was then performed in the same mice using an inVision 128 small animal imaging system (iThera Medical GmbH, Munich, Germany). The mice with the implanted 5-8F orthotopic tumours were scanned at pre (0-h), 4-h, 8-h, and 12-h intervals using transverse slices with a 0.5-mm step from the nasal portion to the cervical portion at wavelengths of 710, 730, 740, 760, 770, 780, 790, 800, 810, and 850 nm for each position. The mice were anaesthetised with 1.6% isoflurane inhalant delivered in 0.8 L of medical air and 0.1 L of oxygen. An adequate depth of anaesthesia was maintained during image acquisition with the mice oriented dorsal side up in the animal holder. The tumours were identified by a live-feed screen preview multispectral signal. Images were reconstructed using the multispectral processing along with ViewMSOT software (iThera Medical GmbH, Munich, Germany) as previously described [22]. The data were optimised using high-resolution (75 μm) back projection reconstruction.

To further investigate the above findings, the mice were scanned by micro-CT using a customised hybrid optical-CT imaging system to obtain the anatomical structure [35]. Anaesthesia was managed using the approach described earlier. Images were acquired with a 2 × 2 binning and 1-s exposure time setting on an EMCCD (electron multiplier charge-coupled device) with an emission filter of 810 nm (XBPA810, Asahi Spectra, Tokyo, Japan) when the lighting turned off. A photograph was then acquired using the EMCCD with an exposure time of 10 ms with the lighting turned on. The photographs helped to locate the mice and register the optical images with the CT volume. Next, 360 frames of CT projections were acquired in cone beam projection mode. The imaging settings were as follows: 120 μm thickness, 120 × 120 μm^2^ base resolution, 40 kV, and 0.8 mA. Reconstruction and volume-rendered 3D images were obtained using 3D Med 4.6 software. Finally, the excised head and neck tissues were embedded in tissue freezing medium (Leica) and stored at − 80 °C. The tissue sections were cut at a thickness of 10 μm, fixed with 10% buffered formalin, and stained with haematoxylin-eosin.

### In vivo MSOT imaging of NPC hypoxia

At 6-week post-implantation, ipsilateral lesions suspicious for nodal metastases were detected in the five orthotopic NPC-bearing mice during monitoring of tumour growth. To investigate the feasibility of imaging NPC hypoxia at an advanced stage, planar FMI was performed to detect primary NPC and lesions suspicious for lymph node metastasis after intravenous injection of CAIX-800. Two-dimensional cross-sectional images were then obtained from the same mice using the above-mentioned MSOT inVision 128 small animal imaging system and protocol. The images were reconstructed by the ViewMSOT software. Maximum intensity projections were obtained after reconstruction. The head and neck tissues were excised for immunopathology.

### Statistical analysis

The data are shown as the mean ± standard deviations. Statistically significant differences between groups were identified using independent two-samples *t* tests. Statistical analysis was conducted using GraphPad Prism 6 (GraphPad software, San Diego, California). *P* value < 0.05 was considered statistically significant.

## Results

### Synthesis and characterisation of CAIX-800

CAIX targeting agent **1** was labelled using an IRDye 800 NHS ester (Supplementary Fig. 1). Conjugation was confirmed to be successful by mass spectroscopy (Supplementary Fig. 2). The purity was determined to be > 95% by high-performance liquid chromatography (Supplementary Fig. 3). CAIX-FITC was synthesised and characterised as reported elsewhere [29].

### Cell targeting ability of CAIX-FITC in vitro

**Fig. 1:**
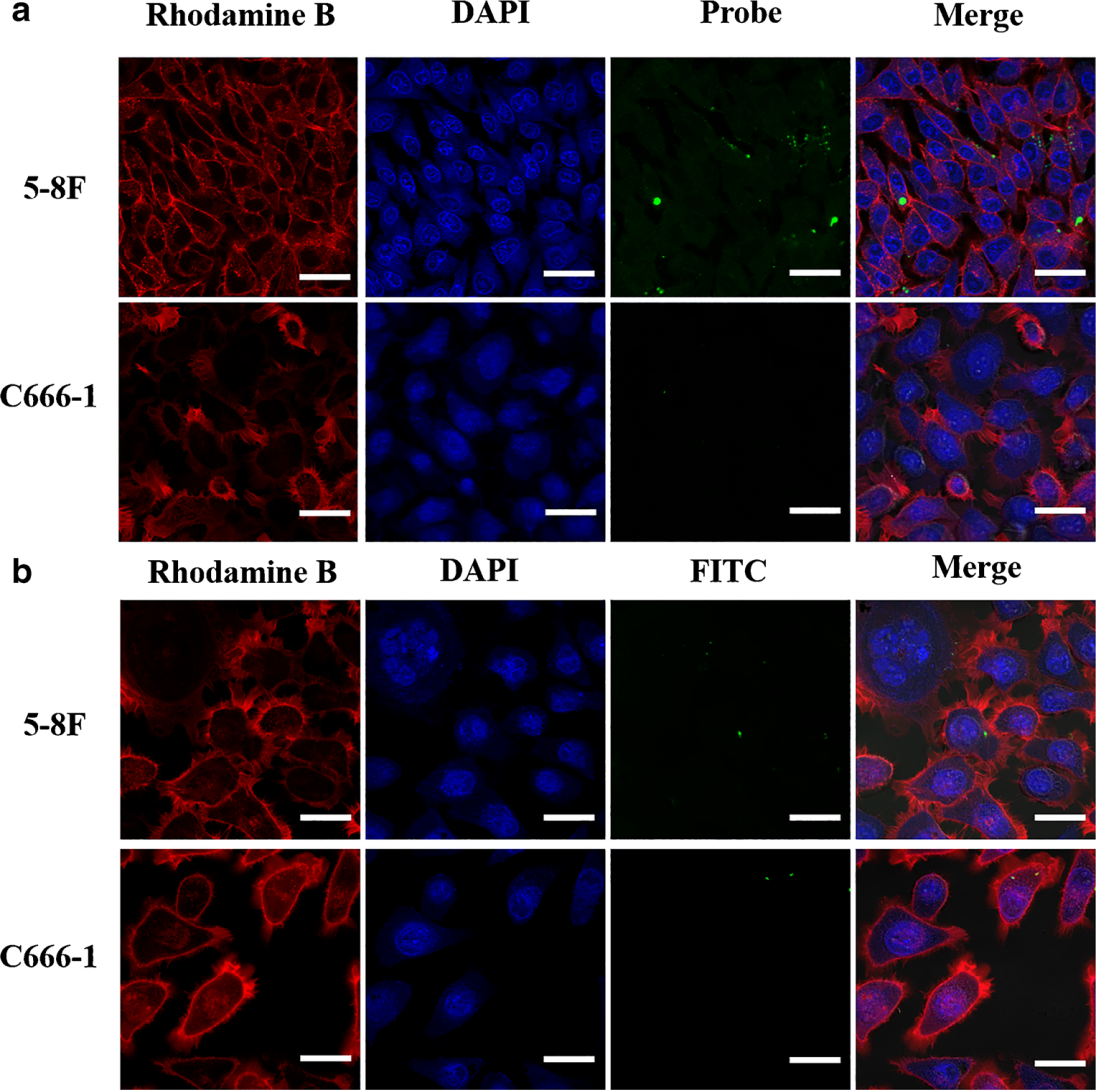
Comparisons of uptake of CAIX-FITC in the different cell lines*.***a** Uptake of CAIX-FITC was markedly higher in 5-8F (CAIX-positive) cells than in C666-1 (CAIX-negative) cells under the same conditions. Scale bar, 20 μm. **b** There was no apparent uptake of free FITC in either 5-8F or C666-1 cells. Scale bar, 10 μm

To validate the targeting efficacy of CAIX, 5-8F (CAIX-positive) and C666–1 (CAIX-negative) cells were fixed and stained with CAIX-FITC to visualise CAIX ligand-induced endocytic trafficking by confocal laser scanning microscopy (Fig. 1). Uptake of CAIX-FITC was much higher in 5-8F cells than in C666-1 cells, and abundant localization of CAIX-FITC was found in the cytomembrane and cytoplasm of 5-8F cells (Fig. 1a). In contrast, there was no apparent uptake of free FITC in either the 5-8F or C666-1 cells in the control groups (Fig. 1b), which confirmed the effective CAIX receptor targeting ability of CAIX-800 in vitro.

### Biodistribution and tumour selectivity of CAIX-800 in vivo

**Fig. 2:**
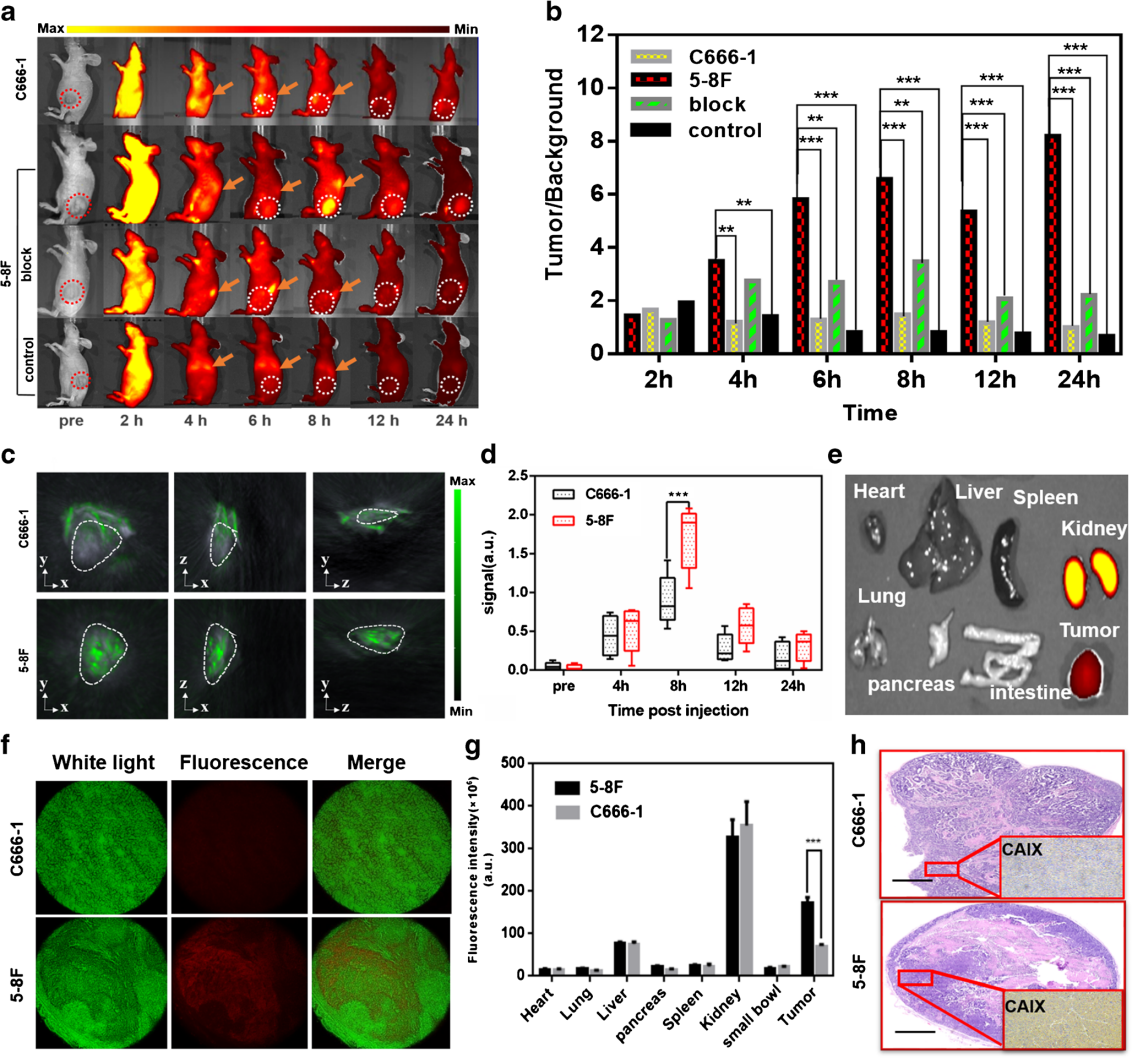
Biodistribution of CAIX-800 in a subcutaneous mouse model of NPC*.***a** Continuous observations of mice bearing subcutaneous C666-1 or 5-8F tumours that received intravenous injections of CAIX-800 using planar FMI for 24 h. The first two rows: C666-1 (CAIX-negative) and 5-8F (CAIX-positive) bearing mice injected with CAIX-800, respectively. The third row: 5-8F bearing mice injected with a blocking dose of free **1** before injecting CAIX-800 (block). The last row: 5-8F bearing mice injected with free dye (control). “Pre” means pre-injection.White dotted circles indicate tumours, and orange arrows show the left kidneys. **b** Quantitative analysis of the tumour-to-background ratio (TBR) was performed in each group (*n* = 5). **c** Three-dimensional volumetric optoacoustic imaging was performed to assess the accumulation of CAIX-800 (pseudo-colour green) in subcutaneous C666-1 and 5-8F tumours at 8-h post-injection. **d** Quantitative comparison of optoacoustic signal at the different time points. **e** An ex vivo fluorescence image of the resected tumour and major organs from a 5-8F tumour-bearing mouse was acquired 24-h post-injection. **f** Different fluorescence intensity levels (pseudo-colour red) in frozen sections (thickness, 10 μm) from C666-1 and 5-8F tumour tissues were evident 24-h post-injection on inverted microscopy. **g** Quantitative comparison of the fluorescence intensity in ex vivo organs and tumours from 5-8F and C666-1 tumour-bearing mice at 24-h post-injection (5 mice per group). **h** Higher expression of CAIX in 5-8F tumours than in C666-1 tumours was also confirmed by immunohistochemical staining. Scale bar, 1 mm. Note: *P < 0.05, **P < 0.01, ***P < 0.001

A biodistribution experiment was performed to evaluate the in vivo tumour uptake of CAIX-800 in the subcutaneous NPC mouse model. Four hours after the i.v. injection with equivalent CAIX-800, the 5-8F group started to show significantly higher probe accumulation and better optical contrast in tumours compared with the C666-1 group (*P* < 0.01 in 4-, 6-, 8-, 12-, and 24-h time points Fig. 2a, b). The highest optical signal in 5-8F tumours was observed 8 h after injection, but the maximum TBR of the 5-8F group reached 8.982 ± 0.85 at the 24-h time point, which was 10.6-fold higher than that of the C666-1 group (maximum TBR = 0.75 ± 0.38). Furthermore, the blocking 5-8F group that received free **1** before injection of CAIX-800 showed significantly less probe accumulation in tumours and a smaller TBR (*P* < 0.01 at 6, 8, 12, and 24 h) than the non-blocking 5-8F group; the control group did not show noticeable tumour uptake of IRDye 800 (Fig. 2a, b). The 3D volumetric optoacoustic imaging findings at 8-h time point were consistent with the planar FMI results (Fig. 2c). Uptake of CAIX-800 in the tumours was significantly higher in the 5-8F group than that in the C666-1 group at 8 h (*P* < 0.001). However, optoacoustic imaging did not identify any significant between-group difference at 4, 12, and 24 h (Fig. 2d).

Ex vivo fluorescence imaging of the organs and tumours resected from the 5-8F tumour-bearing mice revealed high probe accumulation in the kidney and tumour tissue (Fig. 2e), which is in agreement with an earlier report on the use of related radiotracers [29], because low-molecular-weight probes follow renal excretion. This also explained why there was a high contrast of the fluorescence signal in both tumour and kidney areas in the in vivo fluorescence imaging (Fig. 2a, white dotted circles and orange arrows). Near-infrared (NIR) fluorescence microscopy also confirmed that the CAIX-800 signal was stronger in 5-8F tumour specimens than in C666-1 tumour specimens (Fig. 2f). Furthermore, quantitative comparison of the fluorescence intensity in the resected organs and tumours at 24-h time point indicated a significant difference in probe uptake between the 5-8F and C666-1 tumours (*P* < 0.001; Fig. 2g), but there was no other significant difference in biodistribution of the probe. Finally, immunohistochemical staining revealed strong CAIX positivity in the membrane and cytoplasm of 5-8F tumours and negative expression of CAIX in C666-1 tumours.

### NPC-specific multimodality imaging of orthotopic mouse models

**Fig. 3:**
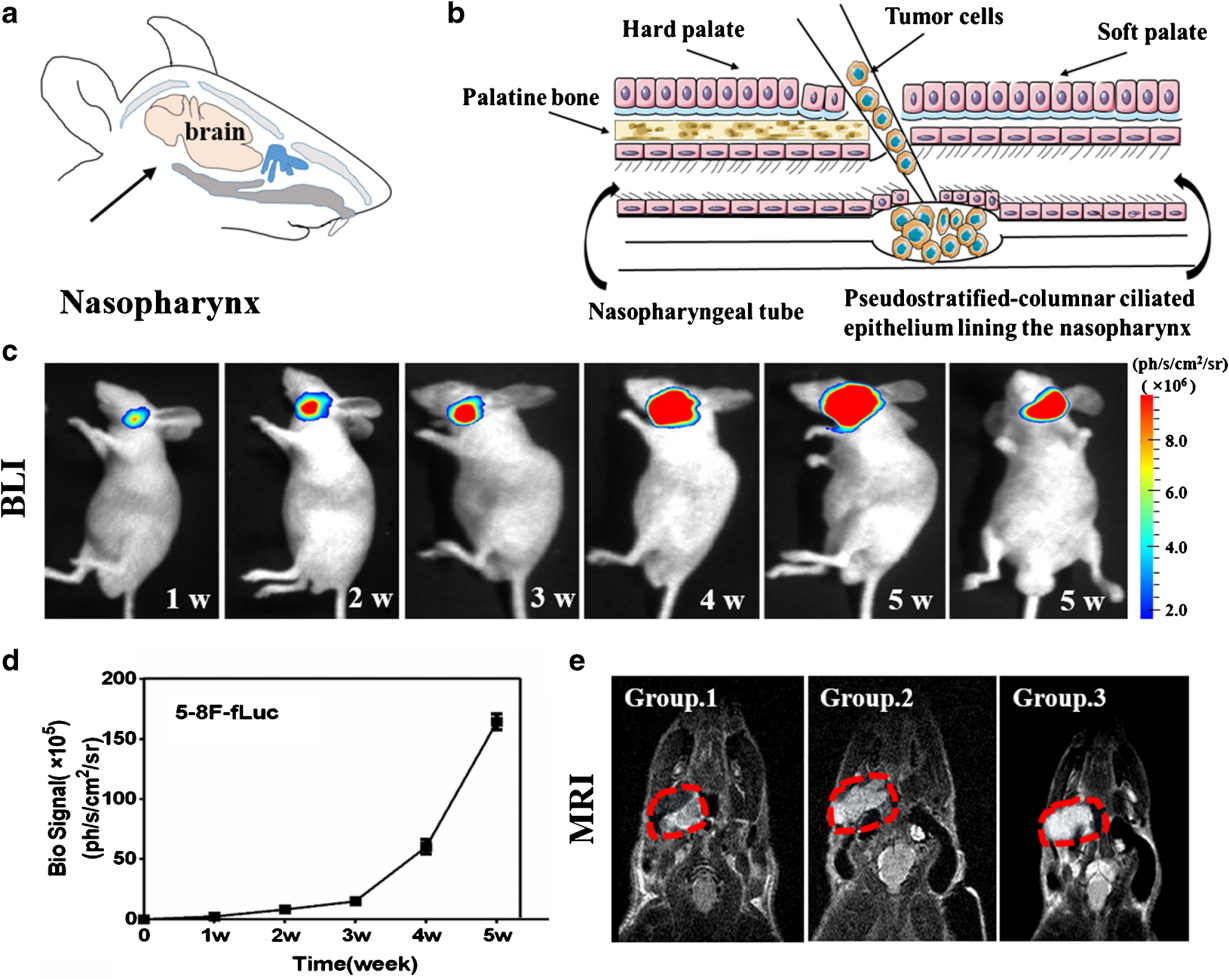
Illustration showing orthotopic NPC implantation. **a** The anatomical location of the nasopharynx (black arrow). **b** 5-8F human NPC cells were injected into the nasopharynx to construct an orthotopic tumour model. **c**) Whole-body bioluminescence imaging was used to track tumour growth. Different sections of the tumour are shown in the right lateral and supine position up to 5 weeks. **d** The mean intensity of the bioluminescent signal recorded from weeks 1–5 post-injection. **e** Tumour locations (dotted red line) were further validated by the coronal T2-weighted MRI 2 weeks after orthotopic injection. With methods of random grouping, the mice were divided into the control group (group 1), the ealry stage of NPC group (group 2), and the advanced stage of NPC group (group 3). Each group had five mice

The orthotopic NPC mouse model was constructed by injecting 5-8F-fLuc cells into the nasopharynx in nude mice (Fig. 3a, b). Growth of the NPC tumours at the orthotopic site was observed by whole-body bioluminescence imaging (PhotonIMAGER; Fig. 3c). A video based on bioluminescence signals showed the tumour location on 3D rendering (Supplementary Video 1), which was reconstructed using a commercial M3 Vision system. The mouse skeleton was simulated by the software without performing CT scanning. Quantitative analysis of tumour growth by weekly measurement of luciferase activity revealed an exponential increase in optical intensity during the following 5 weeks (Fig. 3d). The orthotopic NPC tumours were also confirmed on coronal T2-weighted MRI (Fig. 3e). These results confirmed that we had successfully established an orthotopic mouse model of NPC, lack of which used to be a major obstacle in preclinical research of NPC.

**Fig. 4:**
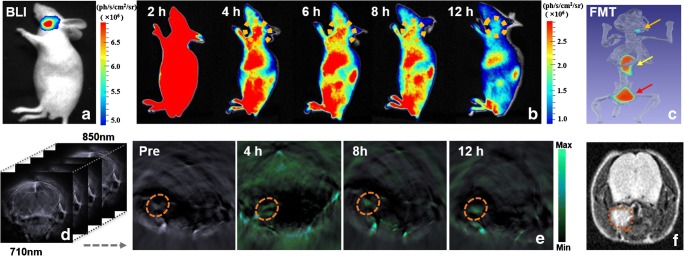
Optical and photoacoustic multimodality imaging of NPC in an orthotopic mouse model. (**a**–**c**) The findings on reference BLI was compared with those on continous FMI observation of the distribution of CAIX-800 in the same orthotopic 5-8F tumour-bearing mouse. Three-dimensional FMT reconstructed by M3 vision shows high probe accumulation in the nasopharynx (orange arrow), kidneys (yellow arrow), and bladder (red arrow). “ph/cm^2^/s/sr” means photon per square centimetre per second per steradian. **d**–**f** Continous observation of the same mouse was also performed using MSOT. Persistent retention of CAIX-800 (pseudo-colour green) in the nasopharynx (orange dotted circles) is clearly visible on tomographic images. The anatomical location was consistent with the reference T2-weighted magnetic resonance image (orange dotted circle). BLI, bioluminescence imaging

The mice with orthotopic NPC were injected with CAIX-800 and scanned using the multimodality imaging approach. Comparison of observations on reference whole-body bioluminescence imaging and on continuous FMI demonstrated that CAIX-800 selectively accumulated in the region of the NPC (Fig. 4a). However, due to the deeper depth of the orthotopic NPC, severe scattering of the fluorescence signal resulted in relatively low contrast between the tumour and the surrounding tissues. In contrast, the FMT reconstruction provided by M3 Vision at the 12-h time point allowed much better visualisation of the distribution of CAIX-800 (Supplementary Video 2), which confirmed that there was indeed selective retention of CAIX-800 in the nasopharynx region. To further validate our findings, we also performed continuous MSOT on the same mice (Fig. 4b). The results demonstrated that the pharmacokinetics of the probe in normal tissue were very different from those in tumour tissue. NPC showed much longer retention of this small optical probe, resulting in clearer tumour contrast from the 8 to 12-h time points. T2-weighted MRI confirmed that MSOT consistently located the tumours.

**Fig. 5:**
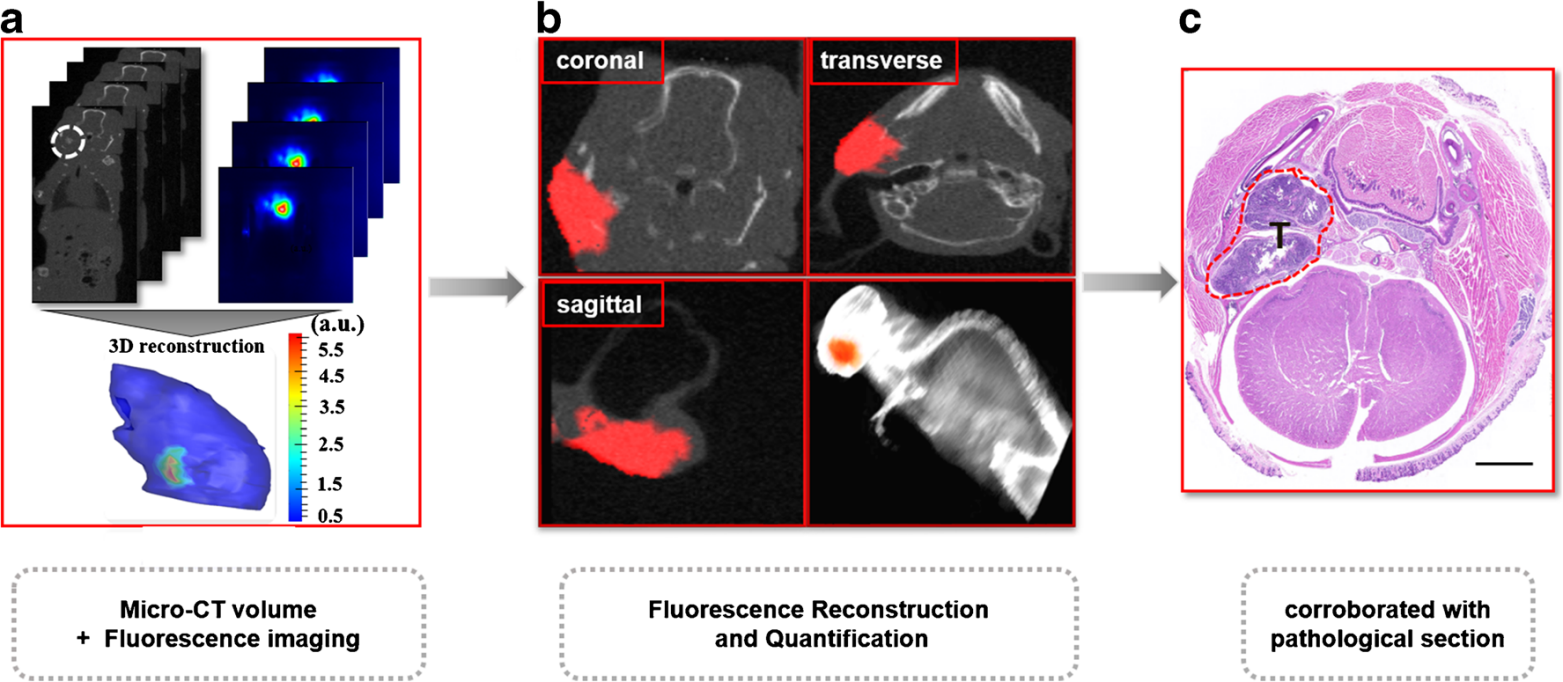
Hybrid FMT-CT imaging of NPC in an orthotopic mouse model. **a** The micro-CT and fluorescence images were both acquired from the same mouse model and merged by 3D mapping. **b** Coronal, transverse, and sagittal views, as well as 3D rendering of the reconstructed fluorescence source inside the CT images, were acquired by fluorescence reconstruction and quantification. **c** Haematoxylin-eosin staining of the corrrsponding cryosection was used for pathological confirmation of the location of the NPC (red dotted contour). Scale bar, 2 mm. 3D, three-dimensional; CT, computed tomography

Given that 3D FMT using the commercial M3 Vision system only provides simulated data, we performed micro-CT and fluorescence imaging hybrid FMT-CT to quantitatively visualise the NPC [36]. The multi-angle NIR fluorescence images and CT images were acquired simultaneously so that the optical flux could be perfectly mapped on the CT data (Fig. 5a). The fluorescent source beneath the skin was reversely reconstructed in 3D (Fig. 5b) [37]. This approach demonstrated an acceptable combination of anatomical structure and the NPC area. FMT-CT showed even higher 3D contrast when compared with MSOT. These imaging results were validated pathologically in haematoxylin-eosin-stained cryosections of orthotopic tumours (Fig. 5c).

### In vivo MSOT imaging of NPC hypoxia

**Fig. 6:**
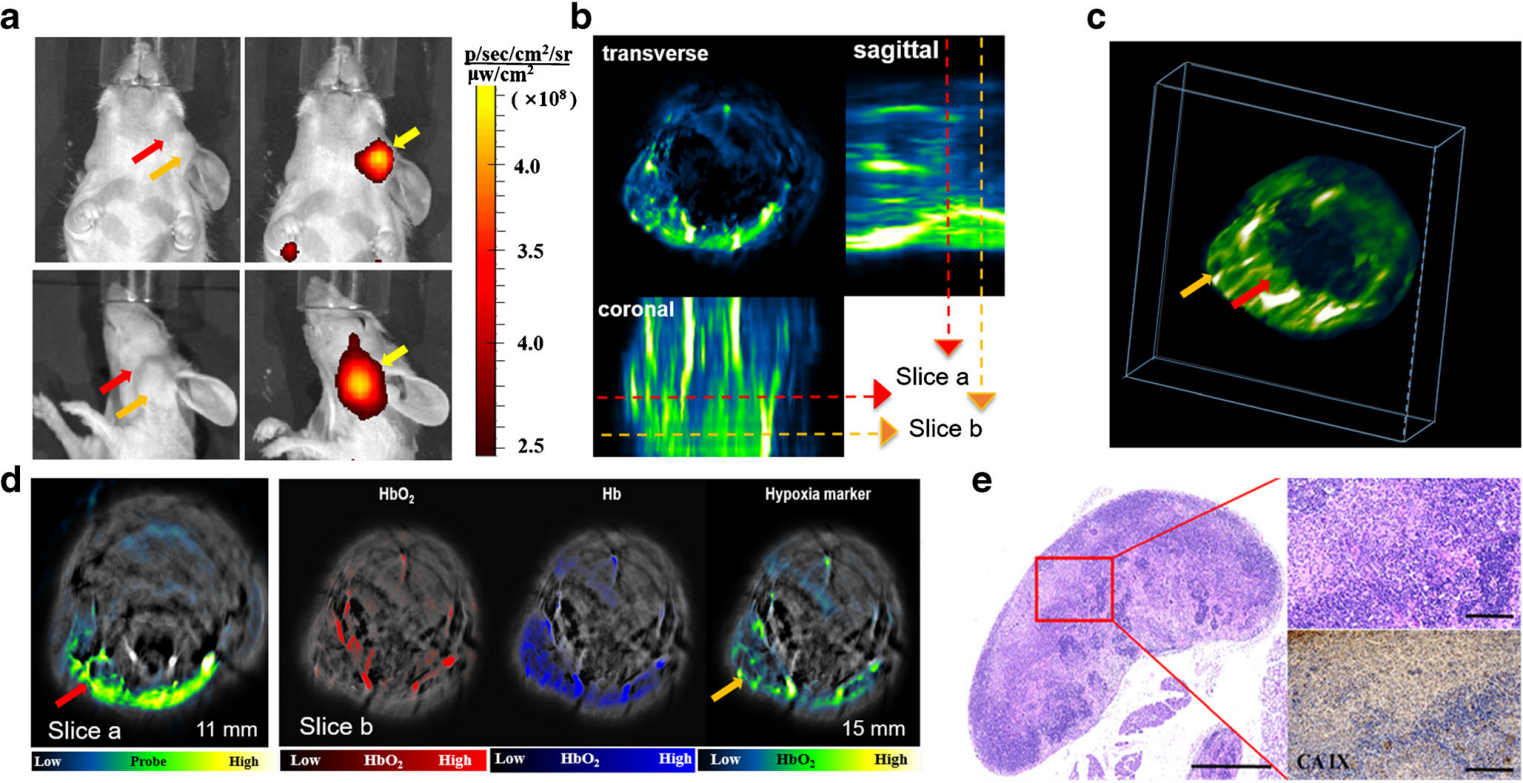
In vivo imaging of hypoxia in NPC by MSOT. **a** Planar fluorescence images show high accumulation of CAIX-800 in the bulged region (yellow arrows) but cannot distinguish between the primary NPC (red arrows) and lymph node metastasis (orange arrows). **b** Transverse, coronal, and sagittal views of the 3D z-stack maximum intensity projection (MIP) post-processed from the MSOT images depict the 3D distribution of CAIX-800. Two slices (red and orange dotted lines) were extracted for comparison. **c** The red and orange arrows indicate the primary NPC and lymph node metastasis on the 3D MIP image,respectively. **d** CAIX-800 enabled visualisation of the primary NPC (red arrow) in slice a, and the multispectral analysis of HbO_2_, Hb, and CAIX-800 allowed comprehensive visualisation of the distribution of hypoxia inside the metastatic node (orange arrow) in slice b. **e** Histological analysis of the enlarged lymph node shows that it is diffusely infiltrated by a population of large atypical cells, confirming the presence of metastatic NPC. Scale bar, 1 mm. Immunohistochemical analysis revealed overexpression of CAIX in the metastatic lymph nodes. Scale bar, 100 μm. CAIX, carbonic anhydrase IX

Six weeks after implantation of the tumours, orthotopic NPC-bearing mice (*n* = 5) spontaneously developed ipsilateral cervical lymph node metastases. Planar FMI showed high accumulation of CAIX-800 in the bulged region (Fig. 6a), but could not differentiate primary NPC from lymph node metastasis because of low spatial resolution caused by optical scattering. Unlike the fluorescence imaging approach, MSOT offered high spatial resolution for observing the distribution of CAIX-800 in 3D (Fig. 6b). Therefore, the primary tumours and lymph node metastases were able to be captured in the rendered 3D image and in different tomographic slices (Fig. 6c, d). Furthermore, the multispectral analysis overlaid the signal of HbO_2_, Hb, and CAIX-800 with the background in MSOT images (Fig. 6d), which provided a comprehensive and quantifiable evaluation of the hypoxic distribution in the tumours. We found that both Hb and CAIX-800 were richly and heterogeneously distributed inside the metastatic lymph nodes whereas HbO_2_ was only concentrated in the peripheral area. Finally, the histological analysis showed that the lymph nodes were diffusely infiltrated by a population of large atypical cells, confirming metastatic NPC, and the immunohistochemical analysis revealed strong expression of CAIX in areas of metastasis (Fig. 6e).

## Discussion

Major improvements in imaging of hypoxia have boosted progress in preclinical research. However, the limitations of single-modality imaging remain a cloud on the horizon [11]. More convincing and accurate data can be obtained if the same probe can be used to target hypoxia in two imaging modes [38, 39]. In this study, we demonstrated that a FMT-CT and MSOT multi-modality imaging strategies was designed for 3D quantitative evaluation of hypoxia in orthotopic NPC-bearing mouse models. We selected CAIX as the molecular marker of NPC hypoxia and synthesised CAIX-800 as the probe for both fluorescence and optoacoustic tomography imaging.

Our experiments on NPC cell uptake in vitro and in a subcutaneous NPC-bearing mouse model in vivo confirmed that CAIX-800 selectively targeted 5-8F tumours but not C666-1 tumours (Figs. 1 and 2) because of the high expression of CAIX in 5-8F cells. Given that CAIX is a well-established biomarker of hypoxia [4, 5, 27, 40, 41], sensitive and accurate 3D imaging of the distribution of CAIX-800 could allow direct evaluation of the level of tumour hypoxia. Therefore, we established an orthotopic NPC-bearing mouse model (Fig. 3) for further investigation.

At the early stage of orthotopic NPC, we performed FMT-CT and MSOT after an intravenous injection of CAIX-800 (Figs. 4 and 5). Compared with conventional 2D FMI, FMT offered much higher NPC contrast and more accurate spatial positioning in 3D, whether simulated by commercial software (Fig. 4a) or reconstructed by our customised hybrid FMT-CT imaging platform (Fig. 5b). FMT is developed to trace back the fluorescent source embedded in the mouse body in an inverse manner, so the fluorescence scattering in animal tissues, which inevitably causes a low TBR on the body surface in 2D FMI, was significantly suppressed for higher contrast [42]. Moreover, although MSOT also detected high accumulation of CAIX-800 in NPC, its tumour contrast does not appear as high as that in FMT-CT (Fig. 4b). This is because the conjugated IRDye 800 is primarily designed for fluorescence imaging, which is normally more sensitive than optoacoustic imaging. The same principle holds true accounting for that in vivo subcutaneous NPC bearing mouse model experiments; 3D volumetric optoacoustic imaging did not find significant difference at the 4-, 12-, and 24-h time points (Fig.2d).Therefore, FMT-CT was a better choice for 3D recognition of NPC hypoxia in the early stage because of its higher sensitivity.

At the advanced stage of orthotopic NPC, the mice developed ipsilateral cervical lymph node metastases. Two-dimensional FMI could not distinguish between the primary tumour and lymph node metastasis (Fig. 6a) because optical imaging loses its spatial resolution dramatically during its propagation in tissues [43]. This could not be resolved by FMT because the surface fluxes from the two fluorescent sources merged into one. However, MSOT allowed more superior visualisation of both the primary tumour and the lymph node metastasis (Fig. 6b–d) because of its high spatial resolution (about 200 μm) [44]. When the NPC progressed into a more advanced stage, the accumulation of CAIX-800 in the tumour was much higher, and the sensitivity of MSOT was no longer bottleneck; at this point, the benefits of high resolution when positioning the primary NPC and metastasis became more apparent. Furthermore, multispectral analysis allowed comprehensive visualisation of the distribution of hypoxia within the metastatic node by combining the information of HbO_2_, Hb, and CAIX-800 (Fig. 6d). The swollen lymph node contained Hb and CAIX-800 but not HbO_2_, indicating severe hypoxia. Multispectral analysis also indicated that the distribution of hypoxia within the metastatic node was markedly heterogeneous. This in vivo observation was confirmed by histological analysis, where infiltration of NPC cells was similarly heterogeneous (Fig. 6e).

Overall, the results of this study demonstrated that integration of FMT-CT and MSOT fully utilised their strengths of sensitivity and resolution in order to achieve a comprehensive and quantifiable evaluation of hypoxia in NPC. In the early stage of NPC, the highly sensitive FMT-CT method enabled precise 3D localization of the hypoxia biomarker with high contrast. In the advanced stage, MSOT enabled multispectral analysis of the biomarker and haemoglobin moleculars with high resolution. To the best of our knowledge, this is the first in vivo study of orthotopic NPC and nodal metastasis that adopted FMT-CT and MSOT for 3D evaluation of hypoxia in different tumour progress stages. Given the ongoing development of various endoscopic instruments for clinical application of NIR fluorescence and optoacoustic imaging [45, 46], our findings have the potential to improve the outcome of radiotherapy in patients with NPC in the future.

Certain limitations and potential biases may exist in our study. Firstly, we focused on the upregulated CAIX as an intrinsic hypoxia-related biomarker, given that the expression of CAIX is dependent on the hypoxic cascade triggered by HIF-1α. However, factors other than hypoxia can also be responsible for its elevated expression [47]. Therefore, future studies should add an exogenous marker, such as pimonidazole, to compensate the potential biases. Both FMT-CT and MSOT have the multiplex imaging ability, and the simultaneous visualisation of multiple biomarkers in NPC is likely to allow more accurate evaluation of hypoxia and more insights on the progression of NPC. Secondly, the lack of comparison of orthotopic NPC mouse models between the different cell lines may have introduced potential bias when evaluating hypoxia. More types of orthotopic NPC mouse models should be included in future studies to better mimic the characteristics of human NPC. Finally, PET-based imaging radiotracers, such as ^18^F-FMISO-PET, would be great for cross-validation with our new method. There should be no spared effort in our future studies.

In conclusion, hypoxia in an orthotopic NPC and lymph node metastasis-bearing mouse model could be quantitatively evaluated in 3D using a FMT-CT and MSOT multimodality imaging strategy. Integration of the high sensitivity and high resolution of these two imaging modalities allowed comprehensive and quantifiable visualisation of hypoxia in different stages of NPC. These findings may potentially benefit patients with NPC undergoing radiotherapy in the future.

## Electronic supplementary material

ESM 1 (DOCX 303 kb)

ESM 2 (AVI 409508 kb)

ESM 3 (AVI 857758 kb)
