# Supplementary material for: In vivo three-dimensional evaluation of tumour hypoxia in nasopharyngeal carcinomas using FMT-CT and MSOT
Source: Eur J Nucl Med Mol Imaging. 2019 Nov 8;47(5):1027–38. doi: 10.1007/s00259-019-04526-x (PMC7101302; doi:10.1007/s00259-019-04526-x)
Supplement: Supplementary file 1 — (DOCX 303 kb) [file 259_2019_4526_MOESM1_ESM.docx]

***Supplementary Figures***

***In vivo* three-dimensional evaluation of hypoxia in nasopharyngeal carcinomas using FMT-CT and MSOT**

**Authors**

Wenhui Huang, Kun Wang, Yu An, Hui Meng, Yuan Gao, Zhiyuan Xiong, Hao Yan, Qian Wang, Xuekang Cai, Bin Zhang, Qiuying, Chen, Xing Yang, Jie Tian, Shuixing Zhang

**Supplementary Fig 1**


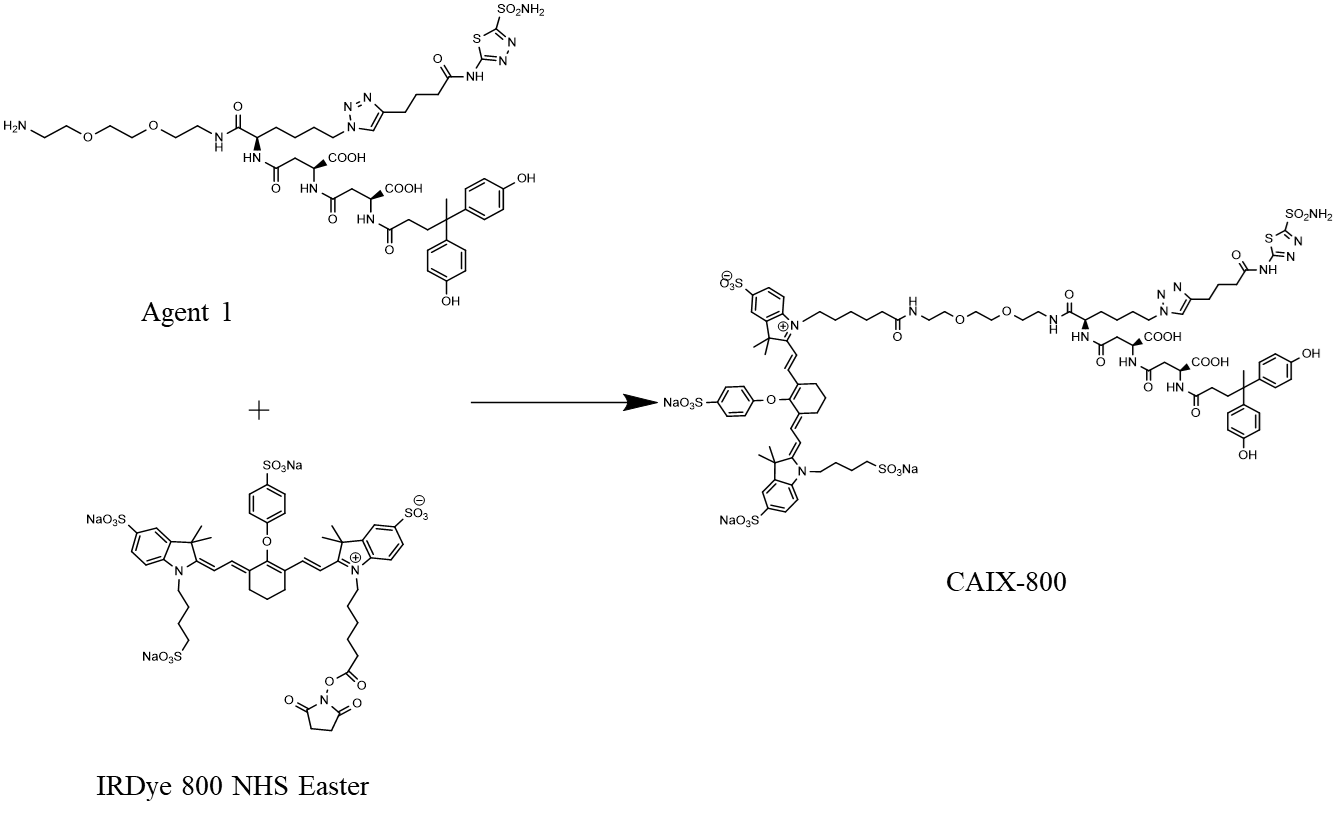


**Supplementary Fig 1.** Synthesis root of CAIX-800.

**Supplementary Fig 2**


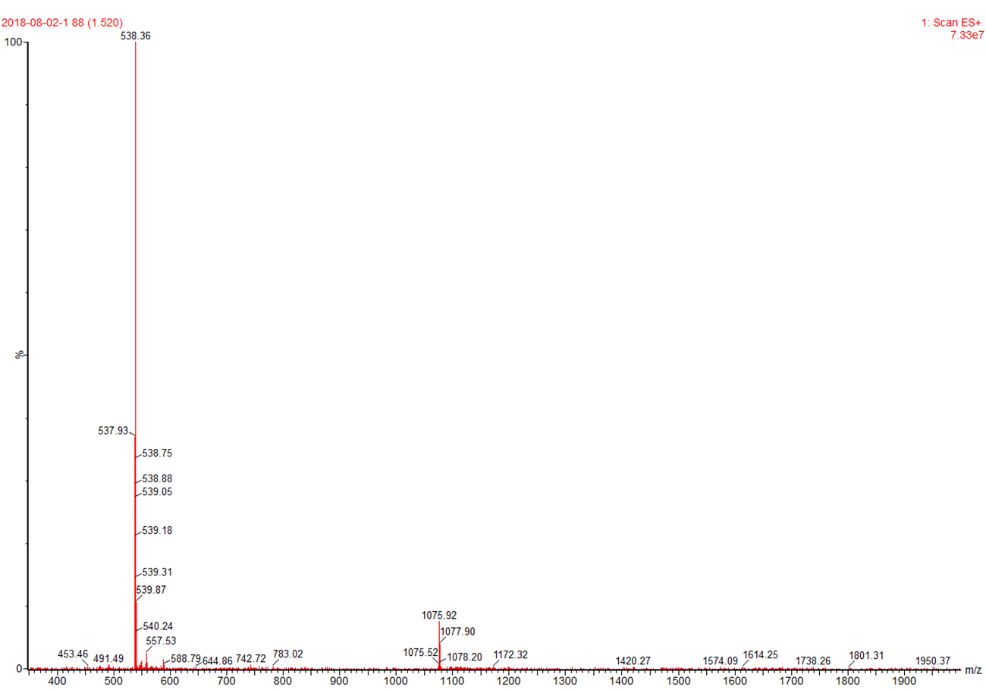


**Supplementary Fig 2.** Corresponding mass spectrometry analysis. HRMS(ESI): m/z 1/4[M+4H+Na]^4+^, calculated for C_91_H_115_N_14_Na_4_O_29_S_6_^4+^: 2151.58; found: 538.36.

**Supplementary Fig 3**


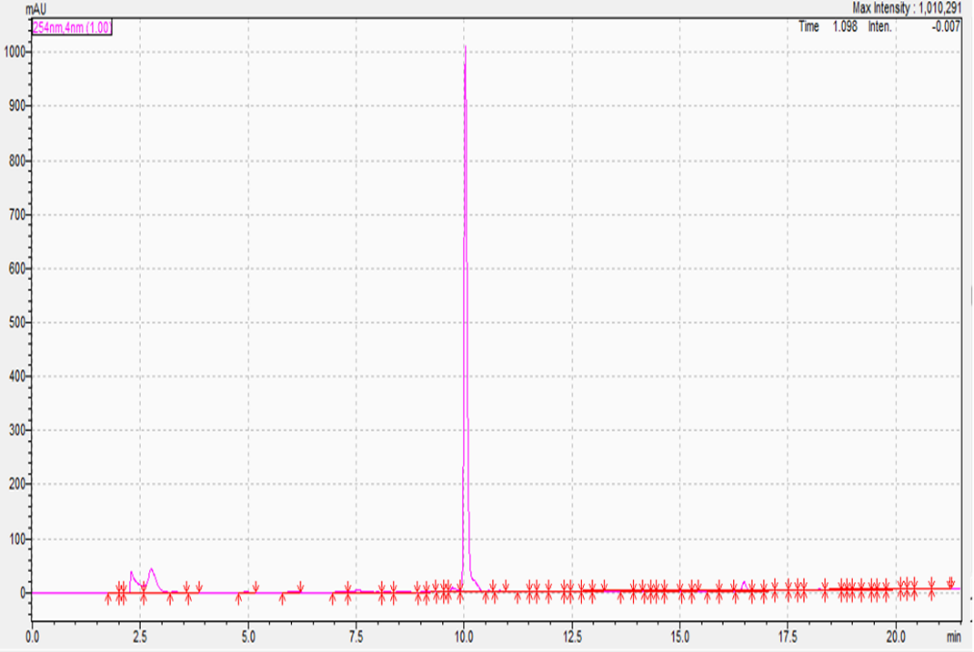


**Supplementary Fig 3**. The HPLC analysis of CAIX-800.
